# Evaluation of Potential DnaK Modulating Proline-Rich Antimicrobial Peptides Identified by Computational Screening

**DOI:** 10.3389/fchem.2022.875233

**Published:** 2022-04-13

**Authors:** Thomas N. G. Handley, Wenyi Li, Nicholas G. Welch, Neil M. O’Brien-Simpson, Mohammed Akhter Hossain, John D. Wade

**Affiliations:** ^1^ The Florey Institute of Neuroscience and Mental Health, University of Melbourne, Parkville, VIC, Australia; ^2^ School of Chemistry, University of Melbourne, Parkville, VIC, Australia; ^3^ ACTV Research Group, Centre for Oral Health Research, Melbourne Dental School, University of Melbourne, Melbourne, VIC, Australia

**Keywords:** PrAMP, antibacterial peptides, DnaK inhibition, *in silico*, solid phase peptide synthesis

## Abstract

The day is rapidly approaching where current antibiotic therapies will no longer be effective due to the development of multi-drug resistant bacteria. Antimicrobial peptides (AMPs) are a promising class of therapeutic agents which have the potential to help address this burgeoning problem. Proline-rich AMPs (PrAMPs) are a sub-class of AMPs, that have multiple modes of action including modulation of the bacterial protein folding chaperone, DnaK. They are highly effective against Gram-negative bacteria and have low toxicity to mammalian cells. Previously we used an *in silico* approach to identify new potential PrAMPs from the DRAMP database. Four of these peptides, antibacterial napin, attacin-C, P9, and PP30, were each chemically assembled and characterized. Together with synthetic oncocin as a reference, each peptide was then assessed for antibacterial activity against Gram-negative/Gram-positive bacteria and for *in vitro* DnaK modulation activity. We observed that these peptides directly modulate DnaK activity independently of eliciting or otherwise an antibiotic effect. Based on our findings, we propose a change to our previously established PrAMP definition to remove the requirement for antimicrobial activity in isolation, leaving the following classifiers: >25% proline, modulation of DnaK AND/OR the 70S ribosome, net charge of +1 or more, produced in response to bacterial infection AND/OR with pronounced antimicrobial activity.

## Introduction

Antimicrobial peptides (AMPs) are an appealing class of naturally occurring, and engineered, peptides with potent and varied antimicrobial activities (Li et al., 2014; Sheard et al., 2019). Moreover, AMPs have distinct modes of activity ranging from membrane permeabilization to enzyme inhibition with some AMPs having multiple differing activities (Raheem and Straus, 2019). Some AMPs are active only against Gram-negative bacteria (Scocchi et al., 2011), which makes them attractive potential antibacterial agents given the level of antibiotic resistance present in such pathogens (Morris and Cerceo, 2020).

Proline rich antimicrobial peptides (PrAMPs) were first reported in 1989 with the discovery of apidaecin in *Apis mellifera* lymph fluid (Casteels et al., 1989). Since then, they have been found in plants (Cao et al., 2015), insects (Casteels et al., 1990; Bulet et al., 1993; Cociancich et al., 1994; Shen et al., 2010), crustaceans (Destoumieux et al., 1997; Stensvåg et al., 2008), and mammals (Agerberth et al., 1991; Mardirossian et al., 2018). In addition to having a direct antimicrobial effect, PrAMPs can also act to inhibit protein synthesis, ultimately contributing to bacterial death, through inhibiting DnaK activity and/or ribosomal translocation through the 70S ribosome (Otvos et al., 2000; Otvos et al., 2001; Krizsan et al., 2014).

Until recently, PrAMPs were classified based on a variety of different criteria depending on the publication (Welch et al., 2020). The definition of a PrAMP was recently updated and confined to those antimicrobial peptides having a 25% or more proline content, a net positive charge, and the capacity to inhibit either DnaK or the 70S ribosome (Welch et al., 2020). This definition does not include an indicator of membrane permeability as PrAMPs are often present as part of an antimicrobial mixture and may gain access to their intracellular target through membrane permeabilization elicited by another peptide (Rabel et al., 2004). Other than membrane permeabilization, PrAMP uptake into the target cell is facilitated primarily through the Gram-negative inner membrane transporters, SbmA and YgdD, although MdtM is also implicated at high PrAMP concentrations (Krizsan et al., 2015; Paulsen et al., 2016; Graf and Wilson, 2019).

Recently, we assessed a new set of criteria for PrAMPs and scanned the DRAMP database (http://dramp.cpu-bioinfor.org/) to detect novel PrAMPs based on similarity to known DnaK inhibitors (Welch et al., 2020). Using these guidelines, eight new peptides were identified as potential new members of the PrAMP family. These are: alpha-defensin-related sequences 7, 10, and 12, antibacterial 6.5 kDa protein, antibacterial napin, attacin C, P9 and PP30. However, this *in silico* identification does not confirm or otherwise the ability to modulate DnaK. Here, we undertook screening of these putative new members for their ability to modulate DnaK activity. A known PrAMP DnaK binder, oncocin (Krizsan et al., 2014), was included in this study as a control. The antibacterial properties of any DnaK modulators were assessed in direct comparison with oncocin to enable objective analysis of the role of DnaK modulation in PrAMP antimicrobial potency. Interestingly, the PRP motif (found in oncocin and other PrAMPs) has been reported to be responsible for binding to DnaK (Zahn et al., 2013). With the potential PrAMPs each lacking this motif, their capacity to modulate, or otherwise, DnaK activity is of interest as it suggests different potential modes of binding. The PRP motif is also absent in other DnaK modulating peptides such as abaecin (Shen et al., 2010), heliocin and formaecin, but each still has significant DnaK inhibition activity (Welch et al., 2020). This confirms that these AMPs bind DnaK at multiple sites (Kragol et al., 2001).

With the current high level of antimicrobial resistance (AMR), the great majority of AMP studies are angled towards eventual potential application in pharmaceutical settings. For PrAMPs to be summarily applied, they need to be simple and cost effective to produce. As such we have selected those putative PrAMPs which lack disulfide bonds and/or cyclic backbones for further investigation. Moreover, we chose not to assess peptides that lack a previously identified, fully defined sequence (i.e. avoiding antibacterial 6.5 kDa protein). The chosen peptides were antibacterial napin, attacin-C, oncocin, P9, and PP30 (Table 1).

**TABLE 1:** Predicted PrAMPs used in this study. The known PrAMPs and control PrAMP, oncocin, is included for reference. Each peptide was synthesized with a C-terminal amide.

Name	Sequence (Single Letter Code)	# AA	%Pro	Net Charge	PRP Motifs	MW Calc	MW Obs
Antibacterial napin	PAQPFRFPKHPQGPQTRPPI	20	35.0	+3	0	2,295.6	2,300.5
Attacin-C	QRPYTQPLIYYPPPPTPPRIYRA	23	34.8	+3	0	2,784.2	2,789.8
Oncocin	VDKPPYLPRPRPPRRIYNR	19	31.6	+5	1	2,389.8	2,395.2
P9	RFIPPILRPPVRPPFRPPFRPPFRPPPIIRFFGG	34	38.2	+7	0	4,034.9	4,040.9
PP30	YVPPVQKPHPNGPKFPTFP	19	36.8	+2	0	2,146.5	2,151.0

## Materials and Methods

### Bacterial Strains

For the antimicrobial assays, the strains used were as follows: *Staphylococcus aureus* ATCC 29213, *Escherichia coli* ATCC 25922 and *E. coli* BAA 3051.

### Peptide Synthesis and Purification

All peptides were synthesized by Fmoc/tBu solid-phase synthesis methods using a Biotage Initiator + Alstra microwave assisted synthesizer, on Rink amide resin with a 0.43 mmol/g loading (RAPP Polymere, Germany) (Li et al., 2017; 2020). Standard Fmoc peptide synthesis protocols were used with 4-fold molar excess of the Fmoc-protected amino acid in the presence of 4-fold O-(1H-6-chlorobenzotriazole-1-yl)-1,1,3,3-tetramethyluronium hexafluorophosphate (HCTU) and 8-fold DIPEA (N, N-diisopropylethylamine). After synthesis, the peptides were cleaved from the solid support using a cleavage cocktail of 95% Trifluoroacetic acid (TFA): 2.5% triisopropylsilane (TIPS): 2.5% H_2_O, for 2 h at room temperature. After cleavage, the resin was removed through a cotton filter and the eluate was transferred to a fresh vessel before evaporating the TFA under a continuous stream of N_2_. The scavengers were removed with two successive washes with ice cold diethyl ether where the ether precipitated the peptide which was collected by centrifugation at 4,000 x G for 5 min before decantation of the ether. The peptides were then purified by reverse-phase high performance liquid chromatography (RP-HPLC) using a Shimadzu LC-20AP with an SPD-20ABLK detector and a column oven at 40 °C with solvents of water and acetonitrile each containing 0.1% TFA, using a gradient of 10–60% acetonitrile in 60 min. The final products were characterized by RP-HPLC over 20 or 30 min from 0 to 100% acetonitrile as well as by matrix-assisted laser desorption/ionization time of flight mass spectrometry (MALDI-TOF MS) on a Shimadzu MALDI-8020 MALDI-TOF mass spectrometer (Supplementary Information). After achieving >95% purity as determined by analytical RP-HPLC, all peptides were exchanged to HCl salt through four rounds of resuspension in 0.2 M HCl followed by freeze drying.

### DnaK Activity Assay

Peptides were assessed for their capacity to inhibit the ATPase activity of DnaK by a colorimetric assay. Peptide at 300 µM concentration were assayed in 100 µl format where each reaction contained 0.3 µM of DnaK in DnaK assay buffer (20 mM TRIS, 150 mM KCl, 5 mM MgCl_2_, 1 mM ATP, pH 7.5). After 60 min, 20 µl samples were taken (3 x per reaction) for assessment by Malachite Green Phosphate assay kit (Sigma—MAK307). The samples were added to 80 µl of Milli-Q water in a 96 well plate, before adding 20 µl of staining solution, and incubated at room temperature for 30 min before measuring A_620_ on a plate reader (Zahn et al., 2013). A negative control was included for each peptide where the same assay conditions are used in the absence of DnaK. The relative activity of DnaK in the presence of each of the putative DnaK binding peptides was assessed using the following equation:

$$DnaKacitivity\;\%=\;\frac{\left(OD_{620}\;sample-OD_{620}\;negative\;control\right)}{\left(OD_{620}\;positive\;control-OD_{620}\;negative\;control\right)}\;\times \;100$$

### Antibacterial Assay

Peptides were assessed for their MIC against various bacterial strains. Peptides were serially diluted at two x final concentration across a 96 well plate with the final well containing no peptide and control lanes of no antibacterial agent, and lanes of no bacteria or antibacterial agent. All dilutions were in sterile MHB. Each bacterial strain was grown overnight before inoculation of a fresh culture in the morning of assay, the culture was allowed to grow until OD_600_ = ∼1.0 before dilution to achieve 2 × 10^6 CFU/ml based of the equations $2e+7e^{\left(2.9218*OD600\right)}$ for *E. coli* and $2e+7e^{\left(2.5537*OD600\right)}.$ Bacteria were added 1:1 to wells containing the serially diluted peptide(s) and were allowed to grow for 6 h, in regards to *E. coli,* or 24 h, in regards to *S. aureus,* before measuring OD_620_ on a plate reader. Bacterial growth per well was determined using the following equation:

$$Bacterial\;growth\;\%=\;\frac{\left(OD_{600}\;sample-OD_{600}\;negative\;control\right)}{\left(OD_{600}\;positive\;control-OD_{600}\;negative\;control\right)}\times 100$$

The MIC breakpoint was determined using GraphPad PRISM observing for the concentration at which growth surpassed 10%.

## Results and Discussion

**FIGURE 1:**
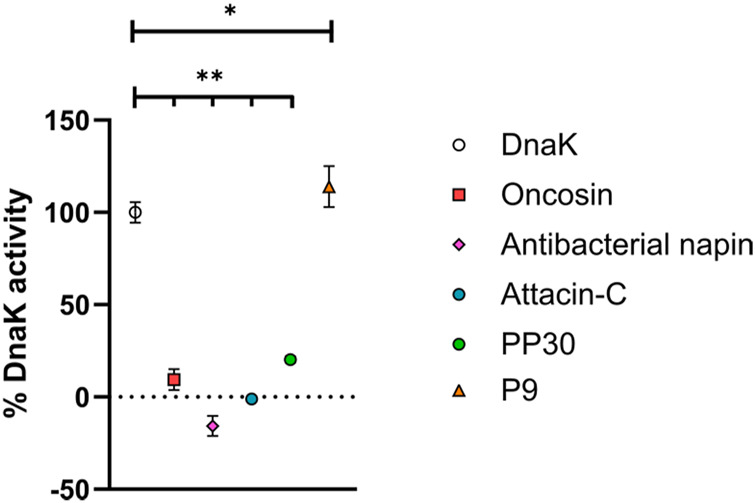
Ability of potential new PrAMPs to impact DnaK activity. Each was incubated with DnaK for 1 h at 37°C and the free phosphate generated was detected with a Malachite Green-based stain. Activities are reported in % activity relative to DnaK positive and DnaK negative samples with constraints at 0% activity. All peptides were assayed at 300 µM. One-way ANOVA followed by Dunnett’s multiple comparisons test was performed to compare the control (DnaK) to each of the peptides, *p* < 0.05 (*) and *p* < 0.001 (**) are indicated.

We selected four potential PrAMPs from the list of newly identified PrAMPs for evaluation. We selected these as they are amenable to ready chemical synthesis, ultimately making them appealing for any downstream pharmaceutical applications (Table 1). Each was chemically synthesized, purified, and exchanged into HCl salt prior to investigation of antimicrobial properties (Supplementary Information, Supplementary Figure S1). The ability of the potential PrAMPs to modulate the activity of DnaK was assessed at 300 µM *in vitro* through a colorimetric assay with a known binder of DnaK, oncocin, as a positive control peptide (Figure 1). It is clear that each of these peptides affected the ATP processing capacity of DnaK but to a greatly varying extent.

The peptides listed in Table 1, antibacterial napin, attacin-C, P9 and PP30, had not been previously reported to interact with DnaK until our most recent study in which computational analysis of AMPs from the DRAMP database was used to identify potential PrAMPs (Welch et al., 2020). As shown in Figure 1, it is clear that our model for predicting DnaK modulating PrAMPs has a 100% success rate where each and every peptide which we have predicted to have an effect on DnaK, in fact do. Antibacterial napin, attacin C, and PP30 each inhibit DnaK activity whereas, somewhat surprisingly, P9 increases the ATP hydrolysis of DnaK. Yet, increasing or decreasing DnaK activity both require interaction with DnaK, although likely through different modes of action. The capacity to accurately identify peptides with the ability to interact with DnaK due to proline content and charge through computational analysis shows promise for narrowing database findings to screen peptides more efficiently for desired characteristics.

The peptides were grouped into two classes: activator (P9), and inhibitors (antibacterial napin, attacin C, and PP30). Interestingly, all these peptides lack PRP motifs. In previous reports, such motifs have been shown to be very important for binding DnaK, with peptides containing multiple PRP motifs having multiple binding modes (Zahn et al., 2013). Our findings of these peptides lacking PRP motifs but still retaining DnaK binding suggest PRP motifs are not the only motif, nor the primary factor, to be able to bind DnaK, with further specific structure activity analysis is required to determine the binding motifs of each of our peptides. This finding is supported by the work of Zahn et al. who identified two other motifs associated with DnaK binding (NRLLLTG and ELPPVKI) (Zahn et al., 2013).

The finding that P9 increased DnaK activity warrants further investigation and mechanistic studies which are outside of the scope of this primary investigation. The effect on bacterial homeostasis with an increase in DnaK activity and associated ATP hydrolysis also needs to be investigated for its potential antimicrobial properties. Increasing DnaK activity may lead to alterations in the protein synthesis pathways within bacteria and ultimately negatively affect their ability to utilize energy sources effectively making them more susceptible to host immune responses. Alternatively, this could improve stress resistance, particularly in response to heat stimuli with DnaK being responsible for the bacterial response to heat. An increase in DnaK activity may promote bacterial resistance to heat stress. As another potential response, the ATP hydrolysis of DnaK may be upregulated without permitting more chaperone-based refolding as a result. If DnaK is trapped in a non-receptive state which is turning over ATP without entering the open conformation which enables refolding of target peptides, it may be realistically inactivated while still using ATP. This would be a double hit to the bacteria as it is not just inactive, it is also utilizing valuable ATP resources.

**FIGURE 2:**
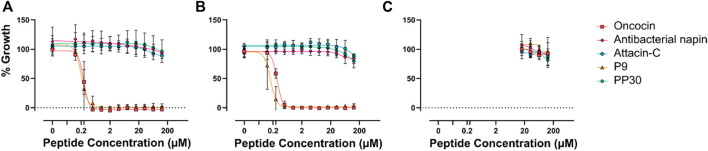
Minimum inhibitory concentration of potential PrAMPs. Each of the potential PrAMPs were assayed for MIC against *E. coli* ATCC 25922 **(A)**, *E. coli* BACC 3051 **(B)**, and *S. aureus* 29213 **(C)**, up to 128 µM in MHB media.

The relationship between the effect on DnaK and antimicrobial potency has not been explored for any of these peptides. Each of these peptides has had some level of antimicrobial testing performed in the past, however, side by side analysis—especially in comparison with oncocin—has not previously been undertaken. Consequently, the relative antimicrobial properties of each of these DnaK modulating peptides was determined (Figure 2).

The MIC data provided interesting insight into the activity of the PrAMPs, particularly P9 which was shown to increase the ATP hydrolysis of DnaK, but in MIC assessment it was shown to have potent antimicrobial activity against *E. coli*. PP30 has previously been shown to be membrane-lytic (Shen et al., 2010). This dual mode of action of PP30, membrane lysis and DnaK activation, is novel and unreported for other native PrAMPs. Interestingly a dimeric form of the artificial PrAMP Chex-Arg20, A3-APO (Szabo et al., 2010), has been shown to be predominantly membrane-lytic (Li et al., 2017; 2022). The dual mode of action of PP30 has not however resulted in observable MICs in the assays here which may be due to a range of factors. We speculate that it is due to the assay conditions of the original research, perhaps due to the specific strains assayed. Nevertheless, *E. coli* was the most responsive of the Gram-negative bacterial strains assayed for MIC (Shen et al., 2010).

It is interesting to note that the peptides tested which inhibit DnaK are in fact not antimicrobial at concentrations of 128 µM or less. This may be because the peptides act *in vivo* as part of a complex multi-peptide cocktail with synergistic effects. Interestingly, antibacterial napin has been shown to be inactive against *E. coli* (Gram-negative bacterium) (Ngai and Ng, 2004), which is corroborated here. However, our observation that it is also inactive against the Gram-positive bacteria *S. aureus* strains tested is in contrast to earlier findings that it is active against a different Gram-positive bacterium *Bacillus subtilis* (Ngai and Ng, 2004)*.* This variation in response to Gram-positive bacterium by antibacterial napin suggests that internalization of the different peptides may vary from species to species.

**FIGURE 3:**
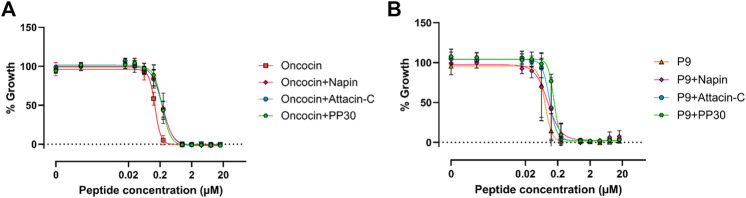
Minimum inhibitory concentrations of antibacterial active and inactive PrAMPs against *E. coli* ATCC 25922. **(A)** MIC assessment of oncocin with/without antibacterial napin/PP30/attacin-C. **(B)** MIC assessment of P9 with/without antibacterial napin/PP30/attacin-C. Each peptide is present in equimolar concentration with the individual concentration shown in the *X* axis.

The lack of direct antimicrobial activity of PP30, attacin-C and antibacterial napin, at realistically achievable therapeutic concentrations on the strains explored here indicates that, although these peptides can modulate DnaK *in vitro*, they do not inhibit *in vivo* bacterial growth of *E. coli* in isolation. One potential reason is that these peptides are unable to access the cytoplasm of the cell. Consequently, the synergistic capacity of PP30, attacin-C, and antibacterial napin with oncocin or P9 was examined (Figure 3). The combination treatment of these peptides showed no synergistic effects. This could be because the peptides are unable to access the cell or because the shared target of DnaK does not allow the peptides to work in synergy. This suggests that co-treatment peptide therapeutic options may be better served with using AMPs with distinct modes of action, as they may be able to act synergistically on different cellular components to improve their potency when compared to their actions when in isolation.

Previously, many PrAMPs have been shown to bind DnaK through their PRP motif(s) which are absent from the PrAMPs explored in this study (excluding the control peptide oncocin). This suggests that PRP is not the only motif that facilitates DnaK binding and, in turn, contribute to antibacterial activity. The PRP motif is needed to bind to the substrate-binding pocket of DnaK (Kragol et al., 2001). While this binding inhibits DnaK protein folding and contributes to killing bacteria, other binding sites on DnaK likely modulate other enzymatic activities that are not necessarily related to antibacterial action. This aspect was highlighted by a recent publication in which 47 analogues of oncosin were evaluated across several different modes of activity including 70S ribosomal binding, inner and outer membrane transport and antimicrobial activity. The lack of clear correlation between these variables led to the conclusion that several parameters, including additional targets such as DnaK, are in continuous play with respect to ultimate antimicrobial action (Brakel et al., 2022; Kolano et al., 2022). Our data confirms this inability to reply solely on one or two *in vitro* assays for the design and prediction of novel antimicrobial peptide analogues. Yet, each of the new PrAMPs assessed here represent interesting and potentially significant new lead compounds in their own right.

## Conclusion

Here we have chemically synthesized, assayed four potential new PrAMP members and confirmed through their interaction with the chaperone DnaK that potential modulators of DnaK can be predicted through computational analysis of antimicrobial peptide databases. However, and importantly, an ability to effect DnaK does not necessarily equate to direct antimicrobial activity. The antimicrobial activity of the PrAMPs tested here, only one of which showed an observable potency, is likely elicited through more complex interaction with other cellular proteins or pathways or in combination with other peptides produced as part of the host defense reaction to a bacterial infection. Considering this change in understanding of antimicrobial activity, we propose an update to the PrAMP definition as follows: Peptides with over 25% proline content, a net charge of +1 or greater, modulation of Dnak AND/OR the 70S ribosome, produced in response to bacterial infection AND/OR with pronounced antimicrobial activity.

## Data Availability

The original contributions presented in the study are included in the article/Supplementary Material, further inquiries can be directed to the corresponding author.

## References

[B1] Agerberth B. Lee J.-Y. Bergman T. Carlquist M. Boman H. G. Mutt V. (1991). Amino Acid Sequence of PR-39. Isolation from Pig Intestine of a New Member of the Family of Proline-Arginine-Rich Antibacterial Peptides. Eur. J. Biochem. 202, 849–854. 10.1111/j.1432-1033.1991.tb16442.x 1765098

[B2] Brakel A. Kolano L. Kraus C. N. Otvos L. Hoffmann R. (2022). Functional Effects of ARV-1502 Analogs against Bacterial Hsp70 and Implications for Antimicrobial Activity. Front. Chem. 10, 798006. 10.3389/fchem.2022.798006 35223768 PMC8864165

[B3] Bulet P. Dimarcq J. L. Hetru C. Lagueux M. Charlet M. Hegy G. (1993). A Novel Inducible Antibacterial Peptide of Drosophila Carries an O-Glycosylated Substitution. J. Biol. Chem. 268, 14893–14897. 10.1016/s0021-9258(18)82417-6 8325867

[B4] Cao H. Ke T. Liu R. Yu J. Dong C. Cheng M. (2015). Identification of a Novel Proline-Rich Antimicrobial Peptide from *Brassica Napus* . Plos One 10, e0137414. 10.1371/journal.pone.0137414 26383098 PMC4575134

[B5] Casteels P. Ampe C. Jacobs F. Vaeck M. Tempst P. (1989). Apidaecins: Antibacterial Peptides from Honeybees. EMBO J. 8, 2387–2391. 10.1002/j.1460-2075.1989.tb08368.x 2676519 PMC401180

[B6] Casteels P. Ampe C. Riviere L. Damme J. Elicone C. Fleming M. (1990). Isolation and Characterization of Abaecin, a Major Antibacterial Response Peptide in the Honeybee (*Apis mellifera*). Eur. J. Biochem. 187, 381–386. 10.1111/j.1432-1033.1990.tb15315.x 2298215

[B7] Cociancich S. Dupont A. Hegy G. Lanot R. Holder F. Hetru C. (1994). Novel Inducible Antibacterial Peptides from a Hemipteran Insect, the Sap-Sucking Bug *Pyrrhocoris apterus* . Biochem. J. 300, 567–575. 10.1042/bj3000567 8002963 PMC1138199

[B8] Destoumieux D. Bulet P. Loew D. Van Dorsselaer A. Rodriguez J. Bachère E. (1997). Penaeidins, a New Family of Antimicrobial Peptides Isolated from the Shrimp *Penaeus Vannamei* (Decapoda). J. Biol. Chem. 272, 28398–28406. 10.1074/jbc.272.45.28398 9353298

[B9] Graf M. Wilson D. N. (2019). “Intracellular Antimicrobial Peptides Targeting the Protein Synthesis Machinery,” in Antimicrobial Peptides. Editor Matsuzaki K. (Springer Singapore), 73–89. 10.1007/978-981-13-3588-4_6 30980354

[B10] Kolano L. Knappe D. Berg A. Berg T. Hoffmann R. (2022). Effect of Amino Acid Substitutions on 70S Ribosomal Binding, Cellular Uptake, and Antimicrobial Activity of Oncocin Onc112. ChemBioChem 23, e202100609. (in press). 10.1002/cbic.202100609 34902208 PMC9306569

[B11] Kragol G. Lovas S. Varadi G. Condie B. A. Hoffmann R. Otvos L. (2001). The Antibacterial Peptide Pyrrhocoricin Inhibits the ATPase Actions of DnaK and Prevents Chaperone-Assisted Protein Folding. Biochemistry 40, 3016–3026. 10.1021/bi002656a 11258915

[B12] Krizsan A. Volke D. Weinert S. Sträter N. Knappe D. Hoffmann R. (2014). Insect-Derived Proline-Rich Antimicrobial Peptides Kill Bacteria by Inhibiting Bacterial Protein Translation at the 70 S Ribosome. Angew. Chem. Int. Ed. 53, 12236–12239. 10.1002/anie.201407145 25220491

[B13] Krizsan A. Knappe D. Hoffmann R. (2015). Influence of the yjiL-mdtM Gene Cluster on the Antibacterial Activity of Proline-Rich Antimicrobial Peptides Overcoming *Escherichia coli* Resistance Induced by the Missing SbmA Transporter System. Antimicrob. Agents Chemother. 59, 5992–5998. 10.1128/aac.01307-15 26169420 PMC4576061

[B14] Li W. Tailhades J. O’Brien-Simpson N. M. Separovic F. Otvos L. Jr. Hossain M. A. (2014). Proline-rich Antimicrobial Peptides: Potential Therapeutics against Antibiotic-Resistant Bacteria. Amino Acids 46, 2287–2294. 10.1007/s00726-014-1820-1 25141976

[B15] Li W. O'Brien-Simpson N. M. Yao S. Tailhades J. Reynolds E. C. Dawson R. M. (2017). C-Terminal Modification and Multimerization Increase the Efficacy of a Proline-Rich Antimicrobial Peptide. Chem. Eur. J. 23, 390–396. 10.1002/chem.201604172 27862429

[B16] Li W. O'Brien-Simpson N. M. Hossain M. A. Wade J. D. (2020). The 9-Fluorenylmethoxycarbonyl (Fmoc) Group in Chemical Peptide Synthesis - its Past, Present, and Future. Aust. J. Chem. 73, 271–276. 10.1071/CH19427

[B17] Li W. Lin F. Hung A. Barlow A. Sani M.-A. Paolini R. (2022). Enhancing Proline-Rich Antimicrobial Peptide Action by Homodimerization: Influence of Bifunctional Linker. Chem. Sci. 13, 2226–2237. 10.1039/D1SC05662J 35310489 PMC8864714

[B18] Mardirossian M. Pérébaskine N. Benincasa M. Gambato S. Hofmann S. Huter P. (2018). The Dolphin Proline-Rich Antimicrobial Peptide Tur1A Inhibits Protein Synthesis by Targeting the Bacterial Ribosome. Cel Chem. Biol. 25, 530–539.e7. 10.1016/j.chembiol.2018.02.004 PMC6219704 29526712

[B19] Morris S. Cerceo E. (2020). Trends, Epidemiology, and Management of Multi-Drug Resistant Gram-Negative Bacterial Infections in the Hospitalized Setting. Antibiotics 9, 196. 10.3390/antibiotics9040196 PMC7235729 32326058

[B20] Ngai P. H. K. Ng T. B. (2004). A Napin-like Polypeptide with Translation-Inhibitory, Trypsin-Inhibitory, Antiproliferative and Antibacterial Activities from Kale Seeds. J. Pept. Res. 64, 202–208. 10.1111/j.1399-3011.2004.00186.x 15485558

[B21] Otvos L. O I. Rogers M. E. Consolvo P. J. Condie B. A. Lovas S. (2000). Interaction between Heat Shock Proteins and Antimicrobial Peptides. Biochemistry 39, 14150–14159. 10.1021/bi0012843 11087363

[B22] Otvos L. Kragol G. Varadi G. Condie B. A. Lovas S. (2001). “The Proline-Rich Antibacterial Peptide Family Inhibits Chaperone-Assisted Protein Folding,” in Peptides: The Wave of the Future. Editors Houghten, R. A. Lebl M. (Springer Netherlands), 873–875. 10.1007/978-94-010-0464-0_408

[B23] Paulsen V. S. Mardirossian M. Blencke H.-M. Benincasa M. Runti G. Nepa M. (2016). Inner Membrane Proteins YgdD and SbmA Are Required for the Complete Susceptibility of *Escherichia coli* to the Proline-Rich Antimicrobial Peptide Arasin 1(1-25). Microbiology 162, 601–609. 10.1099/mic.0.000249 26860543

[B24] Rabel D. Charlet M. Ehret-Sabatier L. Cavicchioli L. Cudic M. Otvos L. (2004). Primary Structure and *In Vitro* Antibacterial Properties of the *Drosophila melanogaster* Attacin C Pro-domain. J. Biol. Chem. 279, 14853–14859. 10.1074/jbc.m313608200 14744858

[B25] Raheem N. Straus S. K. (2019). Mechanisms of Action for Antimicrobial Peptides with Antibacterial and Antibiofilm Functions. Front. Microbiol. 10, 2866. 10.3389/fmicb.2019.02866 31921046 PMC6927293

[B26] Scocchi M. Tossi A. Gennaro R. (2011). Proline-rich Antimicrobial Peptides: Converging to a Non-lytic Mechanism of Action. Cell. Mol. Life Sci. 68, 2317–2330. 10.1007/s00018-011-0721-7 21594684 PMC11114787

[B27] Sheard D. E. O’Brien-Simpson N. M. Wade J. D. Separovic F. (2019). Combating Bacterial Resistance by Combination of Antibiotics with Antimicrobial Peptides. Pure Appl. Chem. 91, 199–209. 10.1515/pac-2018-0707

[B28] Shen X. Ye G. Cheng X. Yu C. Altosaar I. Hu C. (2010). Characterization of an Abaecin-like Antimicrobial Peptide Identified from a Pteromalus Puparum cDNA Clone. J. Invertebr. Pathol. 105, 24–29. 10.1016/j.jip.2010.05.006 20466006

[B29] Stensvåg K. Haug T. Sperstad S. V. Rekdal O. Indrevoll B. Styrvold O. B. (2008). Arasin 1, a Proline-Arginine-Rich Antimicrobial Peptide Isolated from the Spider Crab, Hyas Araneus. Dev. Comp. Immunol. 32, 275–285. 10.1016/j.dci.2007.06.002 17658600

[B30] Szabo D. Ostorhazi E. Binas A. Rozgonyi F. Kocsis B. Cassone M. (2010). The Designer Proline-Rich Antibacterial Peptide A3-APO Is Effective against Systemic *Escherichia coli* Infections in Different Mouse Models. Int. J. Antimicrob. Agents 35, 357–361. 10.1016/j.ijantimicag.2009.10.015 20031377

[B31] Welch N. G. Li W. Hossain M. A. Separovic F. O'Brien-Simpson N. M. Wade J. D. (2020). (Re)Defining the Proline-Rich Antimicrobial Peptide Family and the Identification of Putative New Members. Front. Chem. 8, 607769. 10.3389/fchem.2020.607769 33335890 PMC7736402

[B32] Zahn M. Berthold N. Kieslich B. Knappe D. Hoffmann R. Sträter N. (2013). Structural Studies on the Forward and Reverse Binding Modes of Peptides to the Chaperone DnaK. J. Mol. Biol. 425, 2463–2479. 10.1016/j.jmb.2013.03.041 23562829

