# Supplementary material for: Evaluation of Potential DnaK Modulating Proline-Rich Antimicrobial Peptides Identified by Computational Screening
Source: Front Chem. 2022 Apr 13;10:875233. doi: 10.3389/fchem.2022.875233 (PMC9043238; doi:10.3389/fchem.2022.875233)
Supplement: Supplementary file 1 [file DataSheet1.docx]

**Supplementary Information**

**Evaluation of potential DnaK modulating activity of proline-rich antimicrobial peptides identified by computational screening.**

Thomas N. G. Handley^1,2^, Wenyi Li^3^, Nicholas G. Welch^1^, Neil M. O’Brien-Simpson^3^, Mohammed Akhter Hossain^1,2^ and John D. Wade^1,2*^.

^1^The Florey Institute of Neuroscience and Mental Health, University of Melbourne, Melbourne, VIC 3010, Australia

^2^School of Chemistry, University of Melbourne, Melbourne, VIC 3010, Australia

^3^ACTV Research Group, Centre for Oral Health Research, Melbourne Dental School, University of Melbourne, Melbourne, VIC 3010, Australia

***Correspondence:**John D. Wade, john.wade@florey.edu.au

Supplementary Figure 1: MALDITOF MS trace data for the synthetic peptides reported in this study.

Supplementary Figure 2: RP-HPLC traces of the purified synthetic peptides reported in this study. Each peptide is >95% pure. The retention time in acetonitrile % is reported as ACN RT (in boxes), with the ACN gradient for each RP-HPLC shown in red.
